# Machine learning approaches to identify Parkinson's disease using voice signal features

**DOI:** 10.3389/frai.2023.1084001

**Published:** 2023-03-28

**Authors:** Raya Alshammri, Ghaida Alharbi, Ebtisam Alharbi, Ibrahim Almubark

**Affiliations:** Department of Information Technology, College of Computer, Qassim University, Buraydah, Saudi Arabia

**Keywords:** Parkinson's disease, machine learning, GridSearchCV, SMOTE, feature selection, deep learning

## Abstract

Parkinson's Disease (PD) is the second most common age-related neurological disorder that leads to a range of motor and cognitive symptoms. A PD diagnosis is difficult since its symptoms are quite similar to those of other disorders, such as normal aging and essential tremor. When people reach 50, visible symptoms such as difficulties walking and communicating begin to emerge. Even though there is no cure for PD, certain medications can relieve some of the symptoms. Patients can maintain their lifestyles by controlling the complications caused by the disease. At this point, it is essential to detect this disease and prevent it from progressing. The diagnosis of the disease has been the subject of much research. In our project, we aim to detect PD using different types of Machine Learning (ML), and Deep Learning (DL) models such as Support Vector Machine (SVM), Random Forest (RF), Decision Tree (DT), K-Nearest Neighbor (KNN), and Multi-Layer Perceptron (MLP) to differentiate between healthy and PD patients by voice signal features. The dataset taken from the University of California at Irvine (UCI) machine learning repository consisted of 195 voice recordings of examinations carried out on 31 patients. Moreover, our models were trained using different techniques such as Synthetic Minority Over-sampling Technique (SMOTE), Feature Selection, and hyperparameter tuning (GridSearchCV) to enhance their performance. At the end, we found that MLP and SVM with a ratio of 70:30 train/test split using GridSearchCV with SMOTE gave the best results for our project. MLP performed with an overall accuracy of 98.31%, an overall recall of 98%, an overall precision of 100%, and f1-score of 99%. In addition, SVM performed with an overall accuracy of 95%, an overall recall of 96%, an overall precision of 98%, and f1-score of 97%. The experimental results of this research imply that the proposed method can be used to reliably predict PD and can be easily incorporated into healthcare for diagnosis purposes.

## 1. Introduction

Millions of individuals worldwide are affected by Parkinson's Disease (PD), a progressively deteriorating disorder in which symptoms appear gradually over time. While visible symptoms occur in people over the age of 50, roughly one in every ten people shows signs of this disease before the age of 40 (Marton, 2019). Parkinson's disease causes the death of specific nerve cells in the brain's substantia nigra, which generate chemical dopamine for directing bodily movements. Dopamine deficiency causes additional progressive symptoms to emerge gradually over time. Typically, PD symptoms begin with tremors or stiffness on one side of the body, such as the hand or arm. Individuals with PD may acquire dementia at later stages (Tolosa et al., 2006). From 1996 to 2016, the global prevalence of PD more than quadrupled, from 2.5 million to 6.1 million individuals. Increased life expectancy has resulted in an older population, which explains the substantial rise (Fothergill-Misbah et al., 2020). The brain is the body's controlling organ. Trauma or sickness to any portion of the brain will manifest in a variety of ways in numerous other sections of the body. PD causes a range of symptoms, including partial or complete loss of motor reflexes, speech problems and eventual failure, odd behavior, loss of mental thinking, and other critical skills. It is difficult to distinguish between typical cognitive function losses associated with aging and early PD symptoms. In the United States, the overall economic impact in 2017 was predicted to be $51.9 billion, including an indirect cost of $14.2 billion, non-medical expenditures of $7.5 billion, and $4.8 billion accruing to disability income for owner's public works. The majority of Parkinson's disease patients are over the age of 65, and the overall economic burden is expected to approach $79 billion by 2037 (Yang et al., 2020). The diagnosis of PD in National Collaborating Centre for Chronic Conditions (2006) is typically based on a few invasive techniques as well as empirical testing and examinations. Invasive diagnostic procedures for PD are exceedingly expensive, inefficient, and require extremely complex equipment with poor accuracy. New techniques are needed to diagnose PD. Therefore, less expensive, simplified, and reliable methods should be adapted to diagnose disease and ensure treatments. However, noninvasive diagnosis techniques for PD require being investigated. Machine learning techniques are used to classify people with PD and healthy people. It has been determined that disorders' vocal issues can be assessed for early PD detection (Harel et al., 2004). So, this study attempts to identify Parkinson's disease (PD) by utilizing Machine Learning (ML) and Deep Learning (DL) models to discriminate between healthy and PD patients based on voice signal features, perhaps lowering some of these expenditures.

## 2. Related work

Several researchers have classified Parkinson's disease using various methods. These studies provide a solid foundation for how machine learning can be applied to neurodegenerative diseases in the face of current challenges in Parkinson's disease subclassification, risk assessment, and prognosis using voice signal features. Selection and classification procedures are used in the (Senturk, 2020) diagnosis technique. The feature selection task took into consideration the methodologies of Feature Importance and Recursive Feature Elimination. Artificial neural networks, support vector machines, and classification and regression trees were all utilized in the trials to categorize Parkinson's patients. Performance comparisons of the different techniques revealed that Support Vector Machines with Recursive Feature Elimination outperformed them. With the fewest vocal features necessary to diagnose Parkinson's, 93.84% accuracy was attained. The results of the methods provided by Gil and Manuel (2009) based on artificial neural networks and support vector machines to aid specialists in the diagnosis of Parkinson's disease indicate a high accuracy of about 90%. Das (2010) compared various classification techniques for the purpose of making an accurate Parkinson's disease diagnosis. The paper's objective is to efficiently identify healthy individuals. A comparative study was carried out. There were four different classification schemes used. These are, in order, Decision Trees, Regression, Neural Networks, and DMneural. The performance score of the classifiers was determined using a variety of evaluation techniques. The neural network classifier produces the best outcomes, as determined by the application scores. The neural network's overall classification performance is 92.9%. A deep belief network (DBN) has been used as a successful method to identify Parkinson's disease in the paper by Al-Fatlawi et al. (2016). The deep belief network (DBN), which is used to produce a template match of the voices, has been configured to accept input from a feature extraction procedure. Using two stacked Restricted Boltzmann Machines (RBMs) and one output layer, DBN is employed in this study to categorize Parkinson's illness. To maximize the networks' parameters, two stages of learning must be used. Unsupervised learning, the first stage, uses RBMs to address the issue that can arise from the initial weights' unpredictable initial value. Secondly, the backpropagation technique is employed for the fine tuning as a supervised learning approach. The experimental results are contrasted with various strategies and related work to demonstrate the efficacy of the suggested system. The proposed approach outperforms all other methods in comparison with its 94% total testing accuracy. Rasheed et al. (2020) proposed two classification schemes to improve the accuracy of PD case identification from voice measurements. They began by applying a variable adaptive moment-based backpropagation algorithm to BPVAM, an artificial neural network. The researchers then investigated the use of dimensionality reduction methods such as principal component analysis (PCA) in conjunction with BPVAM to classify the same dataset. The main goal was to improve PD prediction in the early stages by increasing the system's sensitivity to dealing with fine-grained data. The best results were obtained by BPVAM and BPVAM PCA (97.50%), followed by ANN with Levenberg-Marquardt (95.89%). In their (Kadam and Jadhav, 2019) study, they proposed a feature ensemble learning method based on sparse autoencoders to classify healthy people and people with Parkinson's disease using proper representation of vocal and speech datasets. Feature ensemble learning based on the Sparse Autoencoders method achieves the highest sensitivity and specificity of 97.28% and 90%, respectively. The DNN method achieves the highest sensitivity and specificity of 93.59% and 90%, respectively.

## 3. Material and methods

### 3.1. Dataset

The dataset utilized in the research was obtained from the University of Oxford (UO) repository with collaboration from the National Center for Voice, established by Little et al. (2007, 2009), and is available at the UCI Machine Learning Repository (Little, 2008). The original study presented feature extraction methods for general voice disorders The study included voice recordings from 31 people, including 23 people with Parkinson's Disease (PD) (16 males and 7 females) and eight Healthy Controls (HC) (males = 3 and females = 5). The dataset contains 195 records, 24 columns, and as presented in Table 1, a series of biomedical voice measurements. Table 1 is divided into columns that represent each of the voice measurements and rows which represent vocal recordings from individuals (the “name” column). An average of six recordings were made for each patient; six recordings were taken from 22 patients, and seven recordings were taken from nine patients. The patients' ages ranged from 46 to 85 years (mean 65.8, standard deviation 9.8), and the time since diagnosis ranged from 0 to 28 years. Each row corresponds to one voice recording for 36 s. The voice was recorded in an industrial acoustic company sound-treated booth by a microphone placed 8 cm from the mouth and calibrated according to Little et al. (2009). In the dataset, the “status” column is set to 0 for HC and 1 for those with PD, to distinguish healthy individuals from those with PD.

**Table 1:** UCI Dataset used in the research (Little, 2008).

**Voice measure**	**Meaning**
Name	ASCII name of subject and recording number (categorical variables).
MDVP:Fo(Hz)	Average vocal fundamental frequency (Numerical variables).
MDVP:Fhi(Hz)	Maximum vocal fundamental frequency (Numerical variables).
MDVP:Flo(Hz)	Minimum vocal fundamental frequency (Numerical variables).
MDVP:Jitter(%)	
MDVP:Jitter(Abs)	
MDVP: RAP	Several measures of variation in fundamental frequency (Numerical variables).
MDVP: PPQ	
Jitter:DDP	
MDVP:Shimmer	
MDVP:Shimmer(dB)	
Shimmer: APQ3	Several measures of variation in amplitude (Numerical variables).
Shimmer: APQ5	
MDVP: APQ	
Shimmer:DDA	
NHR	Measures of the ratio of noise to tonal components in
HNR	the voice (Numerical variables).
status	0 for HC and 1 for PD (Numerical variables).
RPDE	Nonlinear dynamical complexity measures (Numerical variables).
D2	
DFA	Signal fractal scaling exponent (Numerical variables).
spread1	
spread2	Nonlinear measures of fundamental frequency variation (Numerical variables).
PPE	

### 3.2. Methods

**Figure 1:**
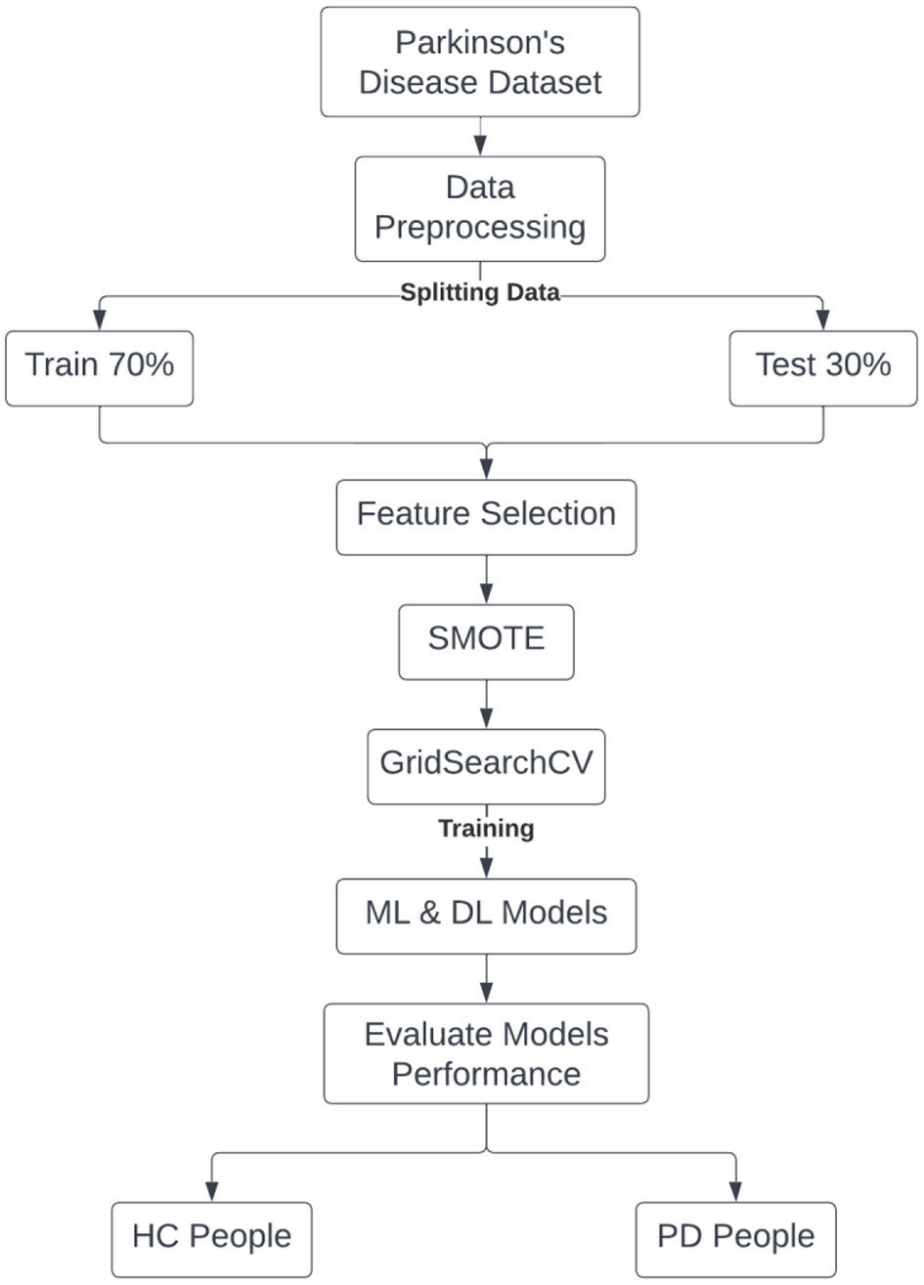
Steps of the proposed classification models.

The proposed method is designed to classify whether the patient has PD or not by using the Google Colab environment and Python language. The methodology of the proposed model is structured into six steps: data preprocessing, features selection, Synthetic Minority Over-sampling Technique (SMOTE), hyperparameter tuning (GridSearchCV), machine and deep learning classification models, and performance evaluation. These steps of the proposed model are shown in Figure 1.

#### 3.2.1. Data preprocessing

Preprocessing is the most important aspect of data processing, which helps the model learn the features of the data effectively and remove unnecessary information (Singh, 2020). The dataset was imported into the Google Colab platform as a CSV file using the Pandas package. After we screened for any duplicates or null entries, we used the “status” column and found that the dataset was imbalanced with 147 for PD and 48 for HC, which is equivalent to 25% for HC and 75% for PD. In order to avoid under-fitting and over-fitting, we split our dataset into a ratio of 70:30 train/test split. The training set includes known outputs, and what the model learns from it may be extended to other data sets. By computing the relevant statistics on the samples in the training set, each feature is scaled individually. The mean and standard deviation are then saved and utilized on later data using the transform in StandardScaler (Teo, 2021). Equation (1) express the mathematical form of StandardScaler normalization. For this study, we employed a variety of libraries, including NumPy, Pandas, Matplotlib, Seaborn, and Sickit-learn (Sklearn). Numpy is Python's fundamental package for scientific computation. It is used to insert any form of mathematical operation into the code. Also, it allows you to include large multidimensional arrays and matrices in your code. The Pandas library is excellent for data manipulation and analysis; it is extensively used for importing and organizing datasets. Matplotlib and Seaborn are the foundations of Python data visualization. Matplotlib is a Python library that can be used to plot 2D graphs with the help of other libraries such as Numpy and Pandas. Seaborn is used to plot graphs using Matplotlib, Pandas, and Numpy. The last one is Sklearn, the most usable and robust machine learning package in Python. It provides a Python-based consistency interface as well as tools for classification, regression, clustering, and dimensionality reduction (Desai, 2019).


(1) $$\begin{array}{l} Standard\;Scaler=\frac{x_{i}-mean\left(x\right)}{stdev\left(x\right)} \end{array}$$

##### 3.2.1.1. Feature selection (FS)

In this phase, SelectKBest was applied to select the eight best features of the dataset. SelectKBest has been found as the second most commonly used dimensionality reduction technique, accounting for 29.1% of total usage (Bilgen et al., 2020). This technique chooses features based on the highest k score, aiding in the removal of less essential data and reducing training time. The eight features used were: MDVP:Fo(Hz), MDVP:Flo(Hz), MDVP:Shimmer, MDVP:APQ, HNR, spred1 spread2, and PPE.

##### 3.2.1.2. Synthetic Minority Over-sampling Technique (SMOTE)

In our dataset, there are fewer HC samples than PD samples. Oversampling samples in the minority class is one way of resolving imbalanced classes. Duplicate instances from the minority class in the training dataset can be used to accomplish this. This may equalize the distribution of classes, but it provides no extra information. SMOTE, or Synthetic Minority Oversampling Technique, is another method for improving minority data based on previous samples. The SMOTE approach builds a linear connection using close features, then selects a new sample from the minority class along that line (Brownlee, 2020).

##### 3.2.1.3. Hyperparameter tuning (GridSearchCV)

The hyperparameters are variables that the user normally specifies when building the machine learning model. To get the best results from the model, we need to use GridSearchCV to discover the optimum hyperparameter values. Grid search is the most basic search algorithm that produces the most accurate predictions. Grid search is simple to conduct in parallel since each trial runs independently without regard for time sequence (Yu and Zhu, 2020). Primarily, it takes arguments i.e., estimator, param grid, cv. Each of the arguments is described as follows:

- Estimator: the estimator object being used.
- Param grid: a list of parameter values and their names.
- cv: an integer represents the folds for a K-fold cross-validation.

#### 3.2.2. Classification models

Following the preceding stages, the desired classifiers were chosen and applied. Deep Learning (DL) and various Machine Learning (ML) algorithms were explored, including K-Nearest Neighbors (KNN), Support Vector Machine (SVM), Decision Tree (DT), Random Forest (RF), and Multi-Layer Perceptron (MLP).

##### 3.2.2.1. K-nearest neighbors (KNN)

The supervised machine learning algorithm KNN is a simple and straightforward technique. The KNN algorithm assumes that related things are located in close proximity. In other words, comparable objects are close to each other (Hossain et al., 2019).

##### 3.2.2.2. Support vector machine (SVM)

Based on recent advances in statistical learning theory, SVM is part of a new generation of learning systems. It is a linear and non-linear data algorithm. It converts the original data into a higher dimension, from which it may create a hyperplane for data separation using support vectors, which are crucial training tuples (Bind et al., 2015).

##### 3.2.2.3. Decision tree (DT)

The DT belongs to the supervised learning algorithm family. Unlike other supervised learning algorithms, the decision tree approach may also be used for regression and classification. Because of its resilience to noise, tolerance for missing information, management of irrelevant redundant predictive attribute values, low processing cost, interpretability, and robust predictors, the DT is one of the most popular and widely used machine learning algorithms (Charbuty and Abdulazeez, 2021).

##### 3.2.2.4. Random forest (RF)

RF is a collection of classifiers based on decision trees. Each tree is built using a bootstrap sample from the data and a candidate set of features chosen at random. It employs both bagging and random variable selection for tree construction. Once the forest has been built, test instances are percolated down each tree, and the trees give class predictions for their particular classes. A random forest's error rate is determined by the strength of each tree and the correlation between any two trees. It may be used to naturally order the relevance of variables in a regression or classification task (Bind et al., 2015).

##### 3.2.2.5. Multilayer perceptron (MLP)

MLP is a feed-forward artificial neural network having three nodes: an input layer, a hidden layer, and an output layer. The input signal that will be processed is received by the input layer. The output layer is responsible for tasks like prediction and classification. The MLP's true computational engine is an arbitrary number of hidden layers sandwiched between the input and output layers. Data in an MLP moves forward from the input to the output layer, comparable to a feed-forward network. The neurons of the MLP are trained using the back propagation learning approach (Abirami and Chitra, 2020).

#### 3.2.3. Performance evaluation

Classifier performance is measured using evaluation measures. We employed the confusion matrix and the classification report in this study. In a confusion matrix, actual class instances are represented as rows, while predicted class occurrences are represented as columns. The four possible outcomes: True Positives (TP), True Negatives (TN), False Positives (FP), and False Negatives (FN) (Jayaswal, 2020).

True Negative (TN): The number of instances correctly classified into negative class.
True Positive (TP): The number of instances correctly classified into the positive class.
False Positive (FP): The number of instances that are misclassified as positive.
False Negative (FN): The number of instances that belong to the target class but are misclassified as negative. According to these four outcomes, we compute the following metrics (classification report).
Accuracy: Is the probability that a diagnostic test will be carried out accurately. Accuracy displays the classification system's total performance.
Sensitivity/Recall: Is a measure for how thorough classifier is; it measures the capacity to identify every positive instance.
Precision: Can be considered a measure of how exact a classifier is. It is described for each class as the proportion of true positives to the total of true and false positives.
F1-Score: It is the precision and recall of the harmonic mean. It takes both false positives and false negatives into account.

## 4. Results and discussion

**Figure 2:**
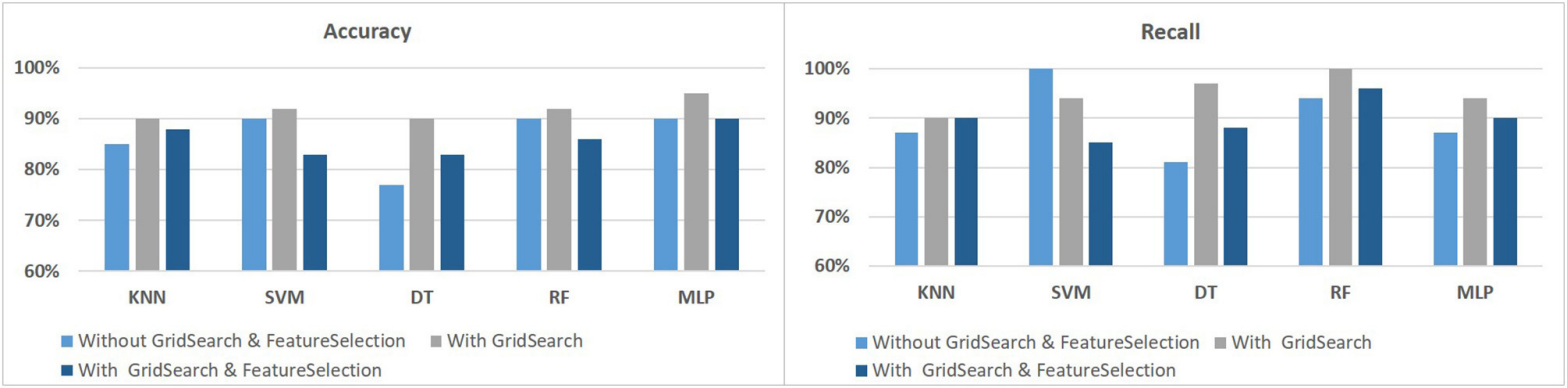
The classification performance without using SMOTE to identify PD using voice signal features.

**Figure 3:**
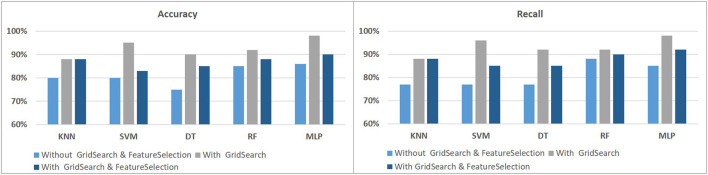
The classification performance using SMOTE to identify PD using voice signal features.

This paper attempts to develop an efficient method of detecting PD by using voice samples. We used the UCI dataset, which contains 195 records of voice signal features collected from 147 PD and 48 HC. We compared different techniques and how they affected our models, although we used various traditional machine learning and deep learning algorithms, such as k-nearest neighbors (KNN), support vector machine (SVM), decision tree (DT), random forest (RF), and multilayer perceptron (MLP). We split the sample into two groups of data in order to demonstrate the performance of the suggested strategy utilizing the dataset. The first category consists of the training samples, which made up 70% of the total samples. The system will be tested, validated, and its accuracy will be checked using the remaining samples. Our dataset appears to be unbalanced because there are much more PD than HC. To address this issue, we used the Synthetic Minority Over-sampling Technique (SMOTE) to balance the dataset. GridSearchCV and SelectKBest were also performed to choose the best features in order to determine the best hyperparameters for our models. The following figures show the results with and without using the SMOTE, as well as with and without GridSearchCV and FeatureSelection. The results in Figures 2, 3 showed that using SMOTE and GridSearch achieved the best performance. However, due to the importance of each feature in the training, we discovered that using FeatureSelection in our dataset did not yield satisfactory results. As a result, we chose to use all features.

According to new findings (Ma et al., 2020), voice dysfunction is the first indicator of motor impairment in PD. Because of the complexity and precision required for vocalization, malfunctions may occur here before the limbs. In perceptual and auditory studies, the voice in Parkinson's disease exhibits distinct changes. So, we are optimistic about the use of voice as a dense biomarker for PD. Our approach exclusively employs voice measurements for clinical diagnosis, as opposed to the most generally acknowledged biomarkers for diagnosis, such as DaT scans or clinician-scored supervised motor assessments in the Unified Parkinson's Disease Rating Scale (UPDRS). Since the voice is one of the first visible signs, we believe that using it will give a faster and more accurate diagnosis than traditional and harmful diagnostic methods, such as handwriting and MRI. Also, the voice diagnosis will be better in terms of low cost, simplicity, and it can be easily incorporated into healthcare. This research diagnoses PD by applying several classification models and comparing their performance to choose the most accurate one.

Table 2 shows the results of the proposed project, and the accurate result was obtained with hyperparameter tuning (GridSearchCV) and SMOTE. Both traditional machine learning (SVM) and deep learning (MLP) algorithms obtained the best results, with 95% and 98.31% accuracy.

**Table 2:** Model's performance.

	**Without gridsearch and feature selection**	**With GridSearch**	**With GridSearch and feature selection**
KNN	Accuracy = 80%	Accuracy = 88%	Accuracy = 88%
	Recall = 77%	Recall = 88%	Recall = 88%
	Precision = 97%	Precision = 98%	Precision = 98%
	F1-Score = 86%	F1-Score = 92%	F1-Score = 92%
SVM	Accuracy = 80%	Accuracy = 95%	Accuracy = 83%
	Recall = 77%	Recall = 96%	Recall = 85%
	Precision = 97%	Precision = 98%	Precision = 93%
	F1-Score = 86%	F1-Score = 97%	F1-Score = 89%
DT	Accuracy = 75%	Accuracy = 90%	Accuracy = 85%
	Recall = 77%	Recall = 92%	Recall = 85%
	Precision = 90%	Precision = 96%	Precision = 95%
	F1-Score = 83%	F1-Score = 94%	F1-Score = 90%
RF	Accuracy = 85%	Accuracy = 92%	Accuracy = 88%
	Recall = 88%	Recall = 92%	Recall = 90%
	Precision = 93%	Precision = 98%	Precision = 96%
	F1-Score = 90%	F1-Score = 95%	F1-Score = 92%
MLP	Accuracy = 86%	Accuracy = 98%	Accuracy = 90%
	Recall = 85%	Recall = 98%	Recall = 92%
	Precision = 98%	Precision = 100%	Precision = 96%
	F1-Score = 91%	F1-Score = 99%	F1-Score = 94%

Numerous researchers used the Parkinson's dataset in classification and regression processes. Table 3 compares the results of this research with those of previous works that made use of the same data. The best performance and accuracy are achieved in this study. In Senturk (2020) they employed three algorithms with feature selection technique, with CART using 7 features and SVM and ANN using 13. The highest result for that paper doesn't exceed 93.84%. Moreover, four algorithms have been applied to classify the data by Das (2010), but their accuracies were below 92.9%, while the accuracy of Gil and Manuel (2009), Al-Fatlawi et al. (2016), Kadam and Jadhav (2019), and Rasheed et al. (2020) reached 97.50% by using neural networks with hyperparameter optimization. Papers in Table 3 did not mention the use of SMOTE to balance the dataset classes except for Gil and Manuel (2009). They mentioned that they improved the accuracy of the classifiers by eliminating a number of outliers from both the minority and majority classes and increasing the size of the minority class to the same size as the majority class. Since the best accuracy obtained was 98.31% using the MLP method, we can conclude that the use of hyperparameter tuning (GridSearchCV) and SMOTE techniques was the most significant factor that contributed to the results of our research. The construction of the MLP architecture proceeds as follows:

1) Layer 1 (input layer) correlates directly to the input vector, which includes all the parameter fields of the patient's record.
2) Layer 2 (hidden layer) the most difficult problem in the network's construction is determining how many hidden neurons are present in this layer. So, we used hyperparameter tuning to save time on experimenting with the best number of neurons, function of network activation, and the algorithm used to improve the network weights (solver). After applying this technique, the optimal value was found to be one hidden layer with 16 neurons, relu activation function, and lbfgs solver.
3) Layer 3 (output layer) is the predictive layer that determine if the patient result is HC or PD, and to check how accurate the model was, we used the classification report.

**Table 3:** Comparison of the accuracy between the proposed work and previous works by applying the same dataset.

**References**	**Method**	**Accuracy**
Senturk (2020)	CART	90.76%
	SVM	93.84%
	ANN	91.54%
Gil and Manuel (2009)	MLP	92.31%
	SVM	93.33%
Das (2010)	Neural network	92.9%
	DMNeural	84.3%
	Regression	88.6%
	DT	84.3%
Al-Fatlawi et al. (2016)	DBN	94%
Rasheed et al. (2020)	BPVAM, BPVAM-PCA	97.50%
Kadam and Jadhav (2019)	DNN	92.19%
	FESA-DNN	93.84%
Proposed work	KNN	88%
	SVM	95%
	DT	90%
	RF	92%
	MLP	98.31%

## 5. Conclusion

In conclusion, we proposed using machine learning and deep learning approaches to Identify Parkinson's Disease by using voice signal features. These methods' results (SVM 95% and MLP 98.31%) are more accurate than previous works. The proposed working model can help in reducing treatment costs by providing initial diagnostics on time. This model can also be used as a teaching tool for medical students and as a soft diagnostic tool for physicians. Also, the accuracy and scalability of this prediction model can both be improved with numerous possible improvements.

## Data availability statement

The original contributions presented in the study are included in the article/supplementary material, further inquiries can be directed to the corresponding authors.

## Author contributions

All authors listed have made a substantial, direct, and intellectual contribution to the work and approved it for publication.
